# Supplementary material for: Evidence-based practice utilization and associated factors among nurses working in Amhara Region Referral Hospitals, Ethiopia
Source: PLoS One. 2021 Mar 19;16(3):e0248834. doi: 10.1371/journal.pone.0248834 (PMC7978364; doi:10.1371/journal.pone.0248834)
Supplement: S1 Checklist — (DOC) [file pone.0248834.s001.doc]

STROBE Statement—Checklist of items that should be included in reports of cross-sectional studies

| **Section/Topic** | Item No | Recommendation | Reported on Page No |
| --- | --- | --- | --- |
| **Title and abstract** | 1 | 1. Indicate the study’s design with a commonly used term in the title or the abstract   *An institution-based cross-sectional study was conducted from March 18 to April 16, 2019; lines 28.* | 2 |
|  |  | 1. Provide in the abstract an informative and balanced summary of what was done and what was found   *The abstract describes the methods and findings; lines 1-25 of abstract section.* | 2 |
| Introduction | | | |
| Background/rationale | 2 | Explain the scientific background and rationale for the investigation being reported  *The background and rationale are described in the Introduction; Lines 1-68 of introduction section.* | 3-5 |
| Objectives | 3 | State specific objectives, including any pre specified hypotheses  *The specific aims of the study are stated in the 9^th^ paragraph line 6-8 of the Introduction section;* | 5 |
| Methods | | | |
| Study design | 4 | Present key elements of study design early in the paper  *The study design is discussed in paragraph 1 line 1 of the methods and materials section.* | 6 |
| Setting | 5 | Describe the setting, locations, and relevant dates, including periods of recruitment, exposure, follow-up, and data collection  *The institutional setting is described in paragraph 1 line 2 of the Methods and materials section;*  *Study locations are described in paragraph 1 line 2-3 of the Methods section; and study timing is stated in paragraphs 1 line 1 of the Methods and material section.* | 6 |
| Participants | 6 | 1. Give the eligibility criteria, and the sources and methods of selection of participants   *Selection of the sample is discussed in paragraph 1 line 7-9 of the Methods and materials section.* | 6 |
| Variables | 7 | Clearly define all outcomes, exposures, predictors, potential confounders, and effect modifiers. Give diagnostic criteria, if applicable  *Outcomes are discussed in the paragraph 4 line 1 and line 3-7 of the Instrument and Measurement part;*  *The predictors are discussed in the paragraph 4 line 2 of the Instrument and Measurement subsection;* | 8 |
| Data sources/ measurement | 8* | For each variable of interest, give sources of data and details of methods of assessment (measurement). Describe comparability of assessment methods if there is more than one group  *Measurement of the outcomes are discussed in the Instrument and Measurement subsection (paragraph 4 line 3-7)* | 8 |
| Bias | 9 | Describe any efforts to address potential sources of bias  *Confounding was determined in the statistical analysis subsection (paragraph 1 line 7)* | 9 |
| Study size | 10 | Explain how the study size was arrived at  *Sample size determination is discussed in paragraph 3 of the Methods section.* | 6 and 7 |
| Quantitative variables | 11 | Explain how quantitative variables were handled in the analyses. If applicable, describe which groupings were chosen and why  *Discussed in the Statistical Analysis subsection* | 9 |
| Statistical methods | 12 | 1. Describe all statistical methods, including those used to control for confounding   *described in the statistical analysis subsection* | 9 |
|  |  | 1. Describe any methods used to examine subgroups and interactions   *no subgroup* | N/A |
|  |  | 1. Explain how missing data were addressed   *no missing data* | N/A |
|  |  | 1. If applicable, describe analytical methods taking account of sampling strategy   *N/A* |  |
|  |  | 1. Describe any sensitivity analyses   *N/A* |  |

| Results | | | |
| --- | --- | --- | --- |
| Participants | 13* | 1. Report numbers of individuals at each stage of study—eg numbers potentially eligible, examined for eligibility, confirmed eligible, included in the study, completing follow-up, and analysed   *Discussed in paragraph 1 of the Results Section.* | 10 |
|  |  | 1. Give reasons for non-participation at each stage   *N/A* | N/A |
|  |  | 1. Consider use of a flow diagram   *N/A* |  |
| Descriptive data | 14* | 1. Give characteristics of study participants (eg demographic, clinical, social) and information on exposures and potential confounders   *Participants’ characteristics are presented in Table 1.* | 11 |
|  |  | 1. Indicate number of participants with missing data for each variable of interest   *There was no missing data* | N/A |
| Outcome data | 15* | Report numbers of outcome events or summary measures  *Table 1: Frequency of EBP utilization among nurses working in ARRH, Ethiopia, 2019* | 12 and 13 |
| Main results | 16 | 1. Give unadjusted estimates and, if applicable, confounder-adjusted estimates and their precision (eg, 95% confidence interval). Make clear which confounders were adjusted for and why they were included   *Discussed in tables 4, 5 and 6* | 15-17 |
|  |  | 1. Report category boundaries when continuous variables were categorized   *There were no continuous variables.* | N/A |
|  |  | 1. If relevant, consider translating estimates of relative risk into absolute risk for a meaningful time period   *There were no estimates of relative risk* | N/A |
| Other analyses | 17 | Report other analyses done—eg analyses of subgroups and interactions, and sensitivity analyses  *There were no subgroups* | N/A |
| **Discussion** | | | |
| Key results | 18 | Summarise key results with reference to study objectives  *Results are summarized in paragraphs 1-11 of the Discussion section* | 18-20 |
| Limitations | 19 | Discuss limitations of the study, taking into account sources of potential bias or imprecision. Discuss both direction and magnitude of any potential bias  *Limitations are discussed in last paragraph of the Discussion section* | 20 |
| Interpretation | 20 | Give a cautious overall interpretation of results considering objectives, limitations, multiplicity of analyses, results from similar studies, and other relevant evidence  *Discussed in the final paragraph of Discussion section and conclusion subsection.* | 20, 21 |
| Generalisability | 21 | Discuss the generalisability (external validity) of the study results  *Discussed in the final paragraph of Discussion section* | 20 |
| Other Information | | | |
| Funding | 22 | Give the source of funding and the role of the funders for the present study and, if applicable, for the original study on which the present article is based  *Cover letter and funding statement* | 15 |

*Give information separately for exposed and unexposed groups.

Note: An Explanation and Elaboration article discusses each checklist item and gives methodological background and published examples of transparent reporting. The STROBE checklist is best used in conjunction with this article (freely available on the Web sites of PLoS Medicine at http://www.plosmedicine.org/, Annals of Internal Medicine at

http://www.annals.org/, and Epidemiology at http://www.epidem.com/). Information on the STROBE Initiative is available at www.strobe-statement.org.
