# Supplementary material for: Evidence-based practice utilization and associated factors among nurses working in Amhara Region Referral Hospitals, Ethiopia
Source: PLoS One. 2021 Mar 19;16(3):e0248834. doi: 10.1371/journal.pone.0248834 (PMC7978364; doi:10.1371/journal.pone.0248834)
Supplement: Attachment — Submitted filename: PONE-D-20-08386_TS.pdf [file pone.0248834.s003.pdf]

PLOS ONE

Evidence-Based Practice Utilization and Associated Factors among Nurses working in Amhara Region Referral Hospitals, Ethiopia.

--Manuscript Draft--

**Manuscript Number:**	PONE-D-20-08386
**Article Type:**	Research Article
**Full Title:**	Evidence-Based Practice Utilization and Associated Factors among Nurses working in Amhara Region Referral Hospitals, Ethiopia.
**Short Title:**	Evidence-Based Practice Utilization and Associated Factors among Nurses
**Corresponding Author:**	Zewdu Bishaw Aynalem, MSc<br>Bahir Dar Health Science College<br>Bahir Dar, Ethiopia, Amhara Region, Bahir Dar ETHIOPIA
**Keywords:**	Evidence-Based Practice, utilization, Nurses, Ethiopia
**Abstract:**	Abstract<br>Background: Applying evidence-based practice during care provision is essential because it improves the quality of care, reduces health care costs and increases patient and family satisfaction. Despite having the above positive effects on patient outcomes, nurses do not consistently utilize evidence-based practice. Hence, this study aimed to assess utilization and associated factors of evidence-based practice among Nurses working in Amhara Region Referral Hospitals, Ethiopia.<br>Methods: An institution based cross-sectional study was conducted from March 18 to April 16, 2019 in Amhara Region Referral Hospitals. A simple random sampling technique was used to select 684 respondents. Data were collected using pretested and self-administered questionnaire. Data were entered into Epi Info version 7.1.2.0 and exported to SPSS version 22.0 for analysis. Bivariable analysis was used primarily and variables with p-value < 0.2 were further examined using multivariable logistic regression model to control con-founders. Then, variables' p-value < 0.05 with 95% CI was used to determine associated factors.<br>Results : From 684 proposed nurses, 671 of them completed the questionnaire giving 98.1% response rate. Of these, 55% (95% CI: 51.2, 58.9) of them had good evidence-based practice utilization. Variables including single (AOR= 1.662: 95% CI: 1.089-2.536), fewer work experience (AOR= 1.849: 95% CI: 1.049-3.257), good knowledge (AOR= 2.044: 95% CI: 1.406-2.972), effective communication skill (AOR= 2.537: 95% CI: 1.744-3.689), EBP training (AOR= 3.224 95% CI: 1.957-5.311), internet access (AOR= 1.655: 95% CI: 1.119-2.448) and evidence-based guideline availability (AOR= 1.827: 95% CI: 1.249-2.673) were found to be predictors of evidence-based practice utilization.<br>Conclusions : The study revealed that evidence-based practice utilization among nurses is low. Availing evidence-based guidelines in the work area, improving facilities internet access and building nurses' evidence-based practice competencies through either by giving separate training or incorporating as part of the curriculum would improve its utilization.<br>Keywords: Evidence-based practice, Nurse, Utilization, Referral Hospital
**Order of Authors:**	Zewdu Bishaw Aynalem, MSc
	Kassahun Gebeyehu Yazew
	Mignote Hailu Gebrie
**Opposed Reviewers:**	Tirunesh Mulugeta Kassa, MPH<br>Bahir Dar University College of Medical and Health Sciences<br>tiru2015mulugeta@gmail.com<br>Due to more exposure in reviewing papers and her background about the subject matter.
	Dejen Getaneh, MSc<br>Head, Department, Debre Tabor University<br>DG198304@yahoo.com<br>field of study

Powered by Editorial Manager® and ProduXion Manager® from Aries Systems Corporation

Additional Information:	
**Question**	**Response**
**Financial Disclosure**<br><br>Enter a financial disclosure statement that describes the sources of funding for the work included in this submission. Review the submission guidelines for detailed requirements. View published research articles from *PLOS ONE* for specific examples.<br><br>This statement is required for submission and **will appear in the published article** if the submission is accepted. Please make sure it is accurate.<br><br>**Unfunded studies**<br>Enter: *The author(s) received no specific funding for this work.*<br><br>**Funded studies**<br>Enter a statement with the following details:<br>• Initials of the authors who received each award<br>• Grant numbers awarded to each author<br>• The full name of each funder<br>• URL of each funder website<br>• Did the sponsors or funders play any role in the study design, data collection and analysis, decision to publish, or preparation of the manuscript?<br>• **NO** - Include this sentence at the end of your statement: *The funders had no role in study design, data collection and analysis, decision to publish, or preparation of the manuscript.*<br>• **YES** - Specify the role(s) played.<br><br>* typeset	Amhara Regional Health Bureau paid per diem for data collectors during data collection. The funder had no role in study design, data collection and analysis, decision to publish, or preparation of the manuscript.
**Competing Interests**<br><br>Use the instructions below to enter a competing interest statement for this submission. On behalf of all authors, disclose any competing interests that could be perceived to bias this work—acknowledging all financial support and any other relevant financial or non-financial competing interests.	The authors have declared that no competing interests exist.

Powered by Editorial Manager® and ProduXion Manager® from Aries Systems Corporation

This statement **will appear in the published article** if the submission is accepted. Please make sure it is accurate. View published research articles from *PLOS ONE* for specific examples.<br><br>**NO authors have competing interests**<br><br>Enter: *The authors have declared that no competing interests exist.*<br><br>**Authors with competing interests**<br><br>Enter competing interest details beginning with this statement:<br><br>*I have read the journal's policy and the authors of this manuscript have the following competing interests: [insert competing interests here]*<br><br>* typeset	
**Ethics Statement**<br><br>Enter an ethics statement for this submission. This statement is required if the study involved:<br><br>• Human participants<br>• Human specimens or tissue<br>• Vertebrate animals or cephalopods<br>• Vertebrate embryos or tissues<br>• Field research<br><br>Write "N/A" if the submission does not require an ethics statement.<br><br>General guidance is provided below. Consult the submission guidelines for detailed instructions. **Make sure that all information entered here is included in the Methods section of the manuscript.**	The study was approved by the School of Nursing ethical review committee on behalf of Institutional review board of University of Gondar. Before beginning data collection, permission letter was obtained from the School of Nursing and was submitted for each Referral Hospital. From each participant, a verbal informed consent was obtained after clearly describing purpose of the study. Again, nurses were informed of their full right to skip or ignore any questions or not to participation from the study.

Powered by Editorial Manager® and ProduXion Manager® from Aries Systems Corporation

**Format for specific study types**<br><br>**Human Subject Research (involving human participants and/or tissue)**<br>• Give the name of the institutional review board or ethics committee that approved the study<br>• Include the approval number and/or a statement indicating approval of this research<br>• Indicate the form of consent obtained (written/oral) or the reason that consent was not obtained (e.g. the data were analyzed anonymously)<br><br>**Animal Research (involving vertebrate animals, embryos or tissues)**<br>• Provide the name of the Institutional Animal Care and Use Committee (IACUC) or other relevant ethics board that reviewed the study protocol, and indicate whether they approved this research or granted a formal waiver of ethical approval<br>• Include an approval number if one was obtained<br>• If the study involved *non-human primates*, add *additional details* about animal welfare and steps taken to ameliorate suffering<br>• If anesthesia, euthanasia, or any kind of animal sacrifice is part of the study, include briefly which substances and/or methods were applied<br><br>**Field Research**<br><br>Include the following details if this study involves the collection of plant, animal, or other materials from a natural setting:<br>• Field permit number<br>• Name of the institution or relevant body that granted permission	
**Data Availability**<br><br>Authors are required to make all data underlying the findings described fully available, without restriction, and from the time of publication. PLOS allows rare exceptions to address legal and ethical concerns. See the PLOS Data Policy and FAQ for detailed information.	Yes - all data are fully available without restriction

A Data Availability Statement describing where the data can be found is required at submission. Your answers to this question constitute the Data Availability Statement and **will be published in the article**, if accepted.<br><br>**Important:** Stating 'data available on request from the author' is not sufficient. If your data are only available upon request, select 'No' for the first question and explain your exceptional situation in the text box.<br><br>Do the authors confirm that all data underlying the findings described in their manuscript are fully available without restriction?	
**Describe where the data may be found in full sentences. If you are copying our sample text, replace any instances of XXX with the appropriate details.**<br><br>• If the data are **held or will be held in a public repository**, include URLs, accession numbers or DOIs. If this information will only be available after acceptance, indicate this by ticking the box below. For example: *All XXX files are available from the XXX database (accession number(s) XXX, XXX.*).<br>• If the data are all contained **within the manuscript and/or Supporting Information files**, enter the following: *All relevant data are within the manuscript and its Supporting Information files.*<br>• If neither of these applies but you are able to provide **details of access elsewhere**, with or without limitations, please do so. For example:<br><br>*Data cannot be shared publicly because of [XXX]. Data are available from the XXX Institutional Data Access / Ethics Committee (contact via XXX) for researchers who meet the criteria for access to confidential data.*<br><br>*The data underlying the results presented in the study are available from (include the name of the third party*	All relevant data are within the manuscript and its Supporting Information files

Powered by Editorial Manager® and ProduXion Manager® from Aries Systems Corporation

and contact information or URL).<br>• This text is appropriate if the data are owned by a third party and authors do not have permission to share the data.<br><br>* typeset	
Additional data availability information:	Tick here if the URLs/accession numbers/DOIs will be available only after acceptance of the manuscript for publication so that we can ensure their inclusion before publication.

Powered by Editorial Manager® and ProduXion Manager® from Aries Systems Corporation

March 22, 2020

Dear Editor

We would like to submit the manuscript entitled “Evidence-Based Practice Utilization and Associated Factors among Nurses working in Amhara Region Referral Hospitals, Ethiopia” by Zewdu Bishaw Aynalem, Kassahun Gebeyehu Yazew, and Mignote Hailu Gebrie to be considered for publication as an original article in the PLOS ONE journal.

Applying evidence-based practice during care provision is essential because it improves the quality of care, reduces health care costs, fills the gap between theories and practice and increases patient and family satisfaction. Despite having the above positive effects on patient outcomes, nurses do not consistently utilize it.

According to the study being singe in marital status, having fewer work experience, good EBP knowledge, taking EBP training, internet access and having evidence-based guideline at the work area were found to be predictor variables to EBP utilization. Also, this study added a new predictor variable, communication skill, and has got a significant relation with EBP utilization.

The findings of this study have implications for nursing practice, education and research. It would help Referral Hospitals to recognize factors related to EBP utilization and help them to take corrective measures to give the most up-to-date, research tested and high-quality patient care. We believe these findings will be of interest to the readers of your journal.

We certify that this manuscript consists of original, unpublished work and is not currently being considered for publication elsewhere. We know of no conflict of interest associated with this publication, and there has been no significant financial support for this work that could have influenced its outcome. I, as corresponding author, confirm that the manuscript has been read and approved for submission by all the named authors.

Sincerely yours.

Zewdu Bishaw Aynalem (corresponding author)

Address: E-mail: zest7@yahoo.com, Phone: +251918088395

Manuscript

Click here to download Manuscript Evidence-Based Practice Utilization @ AF.docx

Evidence-Based Practice Utilization and Associated Factors among Nurses working in Amhara Region Referral Hospitals, Ethiopia.

Zewdu Bishaw Aynalem[1]*, Kassahun Gebeyehu Yazew[2], and Mignote Hailu Gebrie[3]

[1]Department of Nursing, Bahir Dar Health Science College, P.O. Box 684, Bahir Dar, Ethiopia.

[2]Department of Medical Nursing, School of Nursing, College of Medicine and Health Sciences, University of Gondar, P.O. Box 196, Gondar, Ethiopia.

[3]Department of Surgical Nursing, School of Nursing, College of Medicine and Health Sciences, University of Gondar, P.O. Box 196, Gondar, Ethiopia.

***zest7@yahoo.com (ZB)**

Abstract

Background: Applying evidence-based practice during care provision is essential because it improves the quality of care, reduces health care costs and increases patient and family satisfaction. Despite having the above positive effects on patient outcomes, nurses do not consistently utilize evidence-based practice. Hence, this study aimed to assess utilization and associated factors of evidence-based practice among Nurses working in Amhara Region Referral Hospitals, Ethiopia.

Methods: An institution based cross-sectional study was conducted from March 18 to April 16, 2019 in Amhara Region Referral Hospitals. A simple random sampling technique was used to select 684 respondents. Data were collected using pretested and self-administered questionnaire. Data were entered into Epi Info version 7.1.2.0 and exported to SPSS version 22.0 for analysis. Bivariable analysis was used primarily and variables with p-value < 0.2 were further examined using multivariable logistic regression model to control con-founders. Then, variables' p-value < 0.05 with 95% CI was used to determine associated factors.

Results: From 684 proposed nurses, 671 of them completed the questionnaire giving 98.1% response rate. Of these, 55% (95% CI: 51.2, 58.9) of them had good evidence-based practice utilization. Variables including single (AOR= 1.662: 95% CI: 1.089-2.536), fewer work experience (AOR= 1.849: 95% CI: 1.049-3.257), good knowledge (AOR= 2.044: 95% CI: 1.406-2.972), effective communication skill (AOR= 2.537: 95% CI: 1.744-3.689), EBP training (AOR= 3.224 95% CI: 1.957-5.311), internet access (AOR= 1.655: 95% CI: 1.119-2.448) and evidence-based guideline availability (AOR= 1.827: 95% CI: 1.249-2.673) were found to be predictors of evidence-based practice utilization.

Conclusions: The study revealed that evidence-based practice utilization among nurses is low. Availing evidence-based guidelines in the work area, improving facilities internet access and building nurses' evidence-based practice competencies through either by giving separate training or incorporating as part of the curriculum would improve its utilization.

Keywords: Evidence-based practice, Nurse, Utilization, Referral Hospital

Introduction

Evidence-Based Practice (EBP) is an approach to clinical practice whereby clinicians integrate current best evidence with their clinical expertise to make decisions for a specific client by considering his or her preferences and values (1, 2). Current best evidence is about consulting literature to answer a clinical concern. It is about searching various literature and come up with methodologically strongest studies, then using them for practice. As well, as science is dynamic, it is just using latest evidence (3).

Clinical expertise is a knowledge and skill of nurses about their field of study. Nurses have knowledge and skill that are developed from their academic education and clinical experiences. Undoubtedly, nurses have skill about how to communicate with patients and how to elicit their feelings. Thus, it's about integrating their knowledge and skill during care provision (4). Patient preference and value is that patients may arise more concerns during receiving care. Yes, they may arise their religious and spiritual values, socio-cultural values and economic issues, perceptions about health and treatment options. As a result, it is about listening to them and using these as an input during their health care decision making (5).

The nursing profession remains central to utilize EBP (6). Evidence-based practice positively influences the practice of nurses and enables them to shift from tradition to scientific-based practice (7). Similarly, it results in reduced costs, improved patient outcomes and serves as a standard for quality patient care (8, 9), increases patient and family satisfaction and contributes to professional development (10). Again, it results in high job satisfaction (11), increases working efficiency and reduces overtime (12) and fills the gap between theories and practice (13). Even it improves the health care workers' hand hygiene practice in low-resource countries like Uganda and Ethiopia (14, 15).

Despite of having multitude effects on patient outcomes, nurses do not regularly utilize EBP (8). The reason for this is that nurses face numerous challenges in converting evidence into practice (7, 16, 17). Among these; lack of source, lack of time, inadequate skill, lack of training and lack of knowledge took the first (18). By the same token, although, EBP is considered to be the gold standard in nursing practice, studies from different countries have reported that nurses EBP utilization is low: in Norway nurses' practice towards EBP was too a small extent and most nurses

used their experiences, whether good or evil instead of scientific evidence during care provision; in Australia the use of research focused EBP is, one-third, low; in Iran only 41% and in Nepal 49% of nurses used EBP during their health care delivery (19-23).

Similarly, in Africa, studies on utilization of evidence-based practice reported poor: about 54% in Zambia (24), 25.3% in Ghana (25), 30.9% in Nigeria (26) and 12% of nurses in Kenya (27) had utilized research findings during their clinical practice.

In Ethiopia, most of the time, nursing care is not evidence based rather relay on experience, tradition, common sense and untested theories (28, 29). Also, at all levels of the health systems, there is a little culture of using research evidence during decision making (30). Furthermore, previous studies conducted in Amhara Region (31, 32) focused on EBP utilization among all health care professionals which does not specifically showed where the gap is. Therefore, this study was conducted with the aim of determining EBP utilization and identifying factors that affect it among nurses working in Amhara Region Referral Hospitals (ARRH), Ethiopia.

Materials and Methods

Study design, setting and participants

An institution based cross-sectional study was conducted from March 18 to April 16, 2019 among nurses working in ARRH. The Region is located in the North Western and North central part of Ethiopia. It has eleven administrative zones. Currently, the region has sixty-seven public hospitals, five of which; University of Gondar, Felege Hiwot, Dessie, Debre Birhan and Debre Markos are Referral Hospitals. Each Referral Hospital serves 3.5–5 million people (33). During the study period, there were a total of 458, 390, 254, 140 and 223 nurses who assigned in different units of the above mentioned Referral Hospitals respectively. Nurses who had a work experience of 06 months and above were included in the study. However, nurses who were on maternity or annual leave or those who were seriously ill during the data collection period were excluded.

Sample size

For objective one, utilization of evidence-based practice, the sample size was calculated using the single population proportion formula by considering the following assumptions: 95% confidence interval, 51.8% proportion (28), and 5% marginal error. By adding 10% nonresponse rate, the final sample size was 422.

The sample size for the second objective, identifying factors associated with EBP, was also calculated by taking three predictor variables from previous study (31) using Epi Info version 7.1.2.0 software.

Table 1: Sample size calculation for factors associated with utilization of EBP from previous study among nurses working in ARRH, Ethiopia, 2019.

Serial No	Predictor variables	Non-exposed (p1)	Exposed (p2)	Confidence level (%)	Power (%)	Odds ratio	Sample size	10% non-response rate	Final sample size
1.	Knowledge about EBP	42.9	58.7	95	80	1.89	338	34	**372**
2.	Internet access	48.9	60.4	95	80	1.59	622	62	**684**
3.	Self-reported availability of time	43.9	59.9	95	80	1.9	330	33	**363**

The largest sample size from the table was 684, which was higher than the sample size calculated from the single population proportion formula above. Thus, the minimum adequate sample size for this study was **684**.

Sampling procedure

To reach individual nurses, first, we had allocated nurses in the five hospitals proportionally. Then, a list of all nurses was obtained from the hospital human resource. Later, a list of all nurses, from each referral hospital, who had a work experience of six months and above by excluding nurses who are on maternity or annual leave was created and used as a sampling frame. Finally, sample nurses were selected by simple random sampling technique using OpenEpi Version 3 software by considering all nurses working in the same hospital as homogenous concerning EBP utilization **(figure 1).**

Data collection instrument

Data were collected by using a pre-tested, structured and self-administered questionnaire, which was adapted from different literatures (34-38). The questionnaires were prepared in English and organized into six main sections: socio-demographic, knowledge about EBP, attitude towards EBP, communication skill, utilization and associated factors for EBP. Ten BSc nurses and five MSc nurses were recruited as data collector and supervisor respectively. They got a half day training on how to facilitate the data collection process, about the aim of the study, confidentiality and verbal consent. Pre-test was conducted on a similar setting two weeks before the actual data collection time on 10% of the sample size. Supervisors closely followed the data collection process. The principal investigator was made the overall supervision. Every day after data collection, questionnaires were reviewed and checked for completeness. Then, necessary feedback was offered to data collectors in the next morning.

Previous studies proved that the utilization sub scale is valid and reliable. In two studies, its Cronbach's alpha was documented as 0.93 (34) and 0.847 (35). But, for the sake of contextualization, the reliability of the tool was checked via Cronbach's alpha value using the data obtained from the pretest and found to be 0.91, 0.92, 0.85 and 0.92 for knowledge, attitude, utilization and communication sub scales respectively. Similarly, to ensure its validity, the developed tool was given to two experts in the field for critiquing.

The outcome variable of this study was EBP utilization (good/poor) among nurses. The independent variables were sociodemographic characteristics, individual factors and organizational factors. To measure nurses' EBP utilization, six questions were asked. Each

question had a 5-point Likert scale (1 = never, 2 = sometimes, 3 = usually, 4 = often and 5 = always) with a minimum score of 6 and a possible maximum score of 30. According to the Shapiro Wilk test, utilization score was not approximately normally distributed (W statistic = 0.977, df = 671 and p-value < 0.001). Hence, the median {inter quartile range (IQR)} of utilization score, 16 (±9), was used as a cutoff point. Nurses who scored equal and above the cutoff point were categorized as having "good EBP utilization" otherwise "poor EBP utilization".

A total of 17 questions were asked to measure nurses' knowledge about EBP, with each of them having a 3-level response (1= no, 2 = somewhat, 3 = yes). 'No' response was coded as '1', 'somewhat' responses as '2' and 'yes' responses as '3'. Each respondent's total EBP knowledge scores with a possible minimum score of 17 to a maximum 51 were calculated. Using the Shapiro–Wilk test, nurse's knowledge score was checked for normal distribution. Therefore, knowledge about EBP was not normally distributed (W statistic = 0.976, df = 671 and p-value <0.001). The median (IQR) of knowledge score about EBP was 34 (±13). Nurses having a knowledge score equal and above the median score were considered as having "good knowledge" and those below as having "poor knowledge"

Eleven questions were asked to measure nurses' attitude towards EBP. Each attitude question had a five-point rating scale (1= strongly disagree, 2 = disagree, 3 = neutral, 4 = agree and 5= strongly agree). Each respondent had a possible total attitude score ranging from 11 to 55. The normal distribution of respondent's attitude score was checked by Shapiro Wilk test and found not normally distributed (W statistic = 0.888, df =671 and p-value <0.001). The median (IQR) of attitude score towards EBP was 43 (±9). Nurses who scored equal and above the median were labeled as having "favorable attitude" if not as "unfavorable attitude".

Sixteen questions were adapted from a previous study (56) to measure the effectiveness of nurses' communication skill with the patient. Each item had a 5-point Likert scale (1= strongly disagree, 2 = disagree, 3 = neutral, 4 = agree and 5= strongly agree) with a minimum score of 16 and a maximum score of 80. After checking for normal distribution using Shapiro–Wilk test, the median (IQR) communication score, 59 (±25), was set as a cutoff point because communication score was not normally distributed (W statistic = 0.957, df = 671 and p-value < 0.001). Respondents who scored equal and above the median score were classified as having "effective communication" otherwise "ineffective communication".

Statistical analysis

Data were cleaned, coded and entered to Epi-Info version 7.2.1.0 software and then exported to SPSS version 22.0 for analysis. The result was interpreted and presented using appropriate tables, graphs and charts. Descriptive parameters, such as mean, mode and median for continuous variables and frequencies and percentage for categorical variables were calculated. The Binary logistic regression model was fitted to identify factors associated with EBP utilization. Variables with p-value < 0.2 in the bi-variable logistic regression model were furtherly entered into multi-variable logistic regression model to control confounders. Model fitness was checked using a Hosmer-Lemeshow goodness-of-fitness test (p-value = 0.360). Finally, the statistical significance was declared at the p-value less than 0.05 with 95% of Confidence interval (CI).

Ethical approval

The study was approved by the School of Nursing ethical review committee on behalf of Institutional review board of University of Gondar. Before beginning data collection, permission letter was obtained from the School of Nursing and was submitted for each Referral Hospital. From each participant, a verbal informed consent was obtained after clearly describing purpose of the study. Again, nurses were informed of their full right to skip or ignore any questions or not to participation from the study.

Results

Socio-demographic characteristics of the respondents

Out of 684 proposed nurses, 671 of them completed the questionnaire, which makes a response rate of 98.1%. Almost half of nurses 342 (51%) were male. The largest proportion, 442 (65.9%), of them were under the age category of 21-30 years and their median (IQR) age was 29 (±7) years. The majority of the respondents were married 447 (66.6%) and Bachelor's degree holders 571 (85.1%). With regard to education completion: the vast majority, 598 (89.1%), of them completed their education from government institutions whereas the rest were from some private university colleges. The majority of respondents were from surgical and medical units each accounted 27.1% and the least, 6.1%, of them were from the critical care unit. The median (IQR) monthly salary of the nurses was 5294 (±2461) Ethiopian Birr **(Table 2).**

Table 2: Sociodemographic characteristics of nurses working in ARRH, Ethiopia, 2019 (n= 671).

Variables	Category	Numbers	Percent
Sex	Female	329	49
	Male	342	51
	21-30	442	65.9
Age (in years)	31-40	159	23.7
	41-50	58	8.6
	51-60	12	1.8
	Single	224	33.4
Marital status	Married	447	66.6
	Diploma Nurse	62	9.2
Educational status	BSc Nurse	571	85.1
	MSc Nurse	38	5.7
Place of education completion	Government	598	89.1
	Private	73	10.9
	<5	255	38.0
Work experience	5-10	140	20.9
(in years)	>10	116	17.3
	Critical care unit	41	6.1
	Pediatrics unit	74	11
Working unit	Medical unit	182	27.1
	Surgical unit	182	27.1
	Emergency unit	64	9.5
	Ambulatory unit	128	19.1
	<4650	171	25.5
Nurses monthly salary (ETB)	4650 - 5294	175	26.1
	5294 - 7111	199	29.7
	>7111	126	18.8

Individual characteristics

From the total respondent nurses, 517 (77%) of them didn't take EBP training either as part of their curriculum or separately, 42.3% of them had no research experience and 49.5% of them had difficulty in interpreting research findings. As well, about 46.1%, 48.9% and 49.6% of them had poor EBP knowledge, unfavorable attitude towards EBP and ineffective communication skill respectively **(Table 3).**

Table 3: Individual related factors for EBP utilization among nurses working in ARRH, Ethiopia, 2019 (n = 671).

Variables	Category	Number	Percent
Having basic computer skill	Yes	448	66.8
	No	223	33.2
Having research experience	Yes	387	57.7
	No	284	42.3
Being willing to utilize EBP	Yes	526	78.4
	No	145	21.6
Ability of understanding statistical terms	Yes	373	55.6
	No	298	44.4
Ability of interpreting research findings	Yes	339	50.5
	No	332	49.5
Taking EBP course or training	Yes	154	23.0
	No	517	77.0
Knowledge about EBP	Good	362	53.9
	Poor	309	46.1
Attitude towards EBP	Favorable	343	51.1
	Unfavorable	328	48.9
Communication skill	Effective	338	50.4
	Ineffective	333	49.6

Organizational characteristics

Four hundred nine (61%) of nurses reported that evidence-based guidelines are available at the work place. But, nearly half (47.2%) of them reported that there is no internet access to utilize EBP. Similarly, 409 (61%) and 367 (54.7%) nurses reported that there is no enough time and no nurse manager who leads and facilitates EBP utilization at work area respectively **(Table 4)**.

Table 4: Organizational related factors for EBP utilization among nurses working in ARRH, Ethiopia, 2019 (n = 671).

Variables	Category	Number	Percent
Availability of evidence-based guideline	Yes	409	61.0
	No	262	39.0
Internet access	Yes	354	52.8
	No	317	47.2
Computer availability	Yes	307	45.8
	No	364	54.2
Presence of a library with different updated references	Yes	305	45.5
	No	366	54.5
Presence of a nurse manager who leads and facilitates EBP utilization	Yes	304	45.3
	No	367	54.7
Presence of enough time at workplace	Yes	262	39.0
	No	409	61.0
Patient load	≤ 6:1	289	43.1
	> 6:1	382	56.9
Workload	≤39 hours/ week	284	42.3
	>39 hours/week	387	57.7

Evidence-based practice utilization.

In this study, the magnitude of nurses who had good evidence-based practice utilization was found to be 55% (95% CI: 51.2, 58.9). The median (IQR) of nurses' utilization score was 16 (±9).

Factors Affecting evidence-based practice utilization of nurses

A multivariable binary logistic regression analysis was performed to identify factors associated with EBP utilization among nurses. Accordingly, seven variables were found to be statistically associated with EBP utilization after adjusting for confounders. These were marital status, work experience, knowledge about EBP, communication skill, training about EBP, evidence-based guideline availability and internet access.

Nurses who were single in marital status were 66.2% higher (AOR = 1.662: 95% CI: 1.089, 2.536) to have good EBP utilization compared to ever-married nurses. Nurses who had worked for less than 5 years and 5-10 years were about 85% (AOR = 1.849: 95% CI: 1.049, 3.257) and 2.2 times (AOR = 2.227: 95% CI: 1.271, 3.900) more likely to have good EBP utilization compared to those who had worked more than 10 years respectively **(Table 5).**

The odds of having good EBP utilization were 2 times higher among nurses who had good knowledge about EBP (AOR= 2.044: 95% CI: 1.406, 2.972) than those who had poor knowledge. Similarly, those nurses who had effective communication skill were about 2.5 times (AOR= 2.537: 95% CI: 1.744, 3.689) more likely to have good EBP utilization as compared to their contraries.

Nurses who had taken EBP training as part of their curriculum and or separately were about 3.2 times (AOR= 3.224: 95% CI: 1.957, 5.311) more likely to have good EBP utilization compared with those who did not attend it **(Table 6).**

In addition, nurses who had internet access and availability of evidence-based guideline at the work area were 65.5% and 82.7% more likely to have good EBP utilization as compared to their counterparts (AOR= 1.655: 95% CI: 1.119, 2.448) and (AOR= 1.827: 95% CI: 1.249, 2.673) respectively **(Table 7).**

Table 5: Binary logistic regression analyses of socio-demographic characteristics with EBP utilization among nurses working in ARRH, Ethiopia, 2019 (n= 671).

Variables	Categories	EBP Utilization		COR (95%CI)	AOR (95%CI)
		Good N (%)	Poor N (%)		
Sex	Female	189 (28.2%)	140 (20.9%)	1.215 (0.896-1.648)	
	Male	180 (26.8%)	162 (24.1%)	1	
Age (year)	21-30	277 (41.3%)	165 (24.6%)	2.350 (0.734-7.525)	
	31-40	73 (10.9%)	86 (12.8%)	1.188 (0.362-3.903)	
	41-50	14 (2.1%)	44 (6.6%)	0.445 (0.122-1.627)	
	51-60	5 (0.7%)	7(1%)	1	
Marital status	Single	154 (23.0%)	70 (10.4%)	2.374 (1.693-3.329)	1.662 (1.089-2.536)*
	Married	215 (32.0%)	232 (34.6%)	1	1
Level of Education	MSc	35 (5.2%)	3 (0.4%)	16.154 (4.48-58.24)	
	BSc	308 (45.9%)	263 (39.2%)	1.622 (0.954-2.757)	
	Diploma	26 (3.9%)	36 (5.4%)	1	
Place of graduation	Government	336 (50.1%)	262 (39.0%)	1.554 (0.954-2.533)	
	Private	33 (4.9%)	40 (6.0%)	1	
Experience (in years)	<5	203 (30.3%)	122 (18.2%)	4.453 (2.830-7.009)	1.849 (1.049-3.257)*
	5-10	132 (19.7%)	89 (13.3%)	3.970 (2.464-6.396)	2.227 (1.271-3.900)*
	>10	34 (5.1%)	91 (13.6%)	1	1
Working unit	Critical care	24 (3.6%)	17 (2.5%)	1.170 (0.574-2.384)	
	Pediatrics	34 (5.1%)	40 (6.0%)	0.704 (0.396-1.251)	
	Medical	100 (14.9%)	82 (12.2%)	1.010 (0.642-1.592)	
	Surgical	104 (15.5%)	78 (11.6%)	1.105 (0.701-1.742)	
	Emergency	37 (5.5%)	27(4.0%)	1.135 (0.619-2.081)	
	Ambulatory	70 (8.6%)	58 (10.4%)	1	
Average monthly salary (ETB)	> 7111	55 (8.2%)	71 (10.6%)	0.499 (0.313-0.796)	
	5294 -7111	114 (17.0%)	85 (12.7%)	0.864 (0.570-1.310)	
	4650 -5294	96 (14.3%)	79 (11.8%)	0.783(0.510-1.201)	
	< 4650	104 (15.5%)	67 (10.0%)	1	

Where: 1 = Reference, ** = p< 0.001, * = p< 0.05

Table 6: Binary logistic regression analyses of individual factors to utilization of evidence-based practice, among nurses working in ARRH, Ethiopia, 2019 (n= 671).

**Variables**	**EBP Utilization**			
	**Good N (%)**	**Poor N (%)**	**COR (95%CI)**	**AOR (95%CI)**
Knowledge about EBP				
Good	253 (37.7%)	109 (16.2%)	3.862 (2.800-5.327)	2.044 (1.406-2.972)**
Poor	116 (17.3%)	193 (28.8%)	1	1
Attitude towards EBP				
Favorable	210 (31.3%)	133 (19.8%)	1.678 (1.235-2.281)	0.943 (0.637-1.396)
Unfavorable	159 (23.7%)	169 (25.2%)	1	1
Nurse-patient communication				
Effective	232(34.6%)	106 (15.8%)	3.131 (2.281-4.299)	2.537 (1.744-3.689)**
Ineffective	137 (20.4%)	196 (29.2%)	1	1
Having basic computer skill				
Yes	275 (41.0%)	173 (25.8%)	2.181 (1.573-3.025)	0.805 (0.517-1.252)
No	94 (14.0%)	129 (19.2%)	1	1
Having research experience				
Yes	253 (37.7%)	134 (20.0%)	2.734 (1.994-3.750)	1.301 (0.873-1.939)
No	116 (17.3%)	168 (25.0%)	1	1
Ability of understanding statistical terms				
Yes	246 (36.7%)	127 (18.9%)	2.756 (2.012-3.775)	1.066 (0.697-1.633)
No	123 (18.3%)	175 (26.1%)	1	1
Ability of interpreting research findings				
Yes	230 (34.3%)	109 (16.2%)	2.930 (2.137-4.016)	1.362(0.935-1.985)
No	139 (20.7%)	193 (28.8%)	1	1
Taking EBP course or training				
Yes	125 (18.6%)	29 (4.3%)	4.823 (3.108-7.483	3.224 (1.957-5.311)**
No	244 (36.4%)	273 (40.7%)	1	1

Where: 1 = Reference, ** = p< 0.001, * = p< 0.05

Table 7: Binary logistic regression analyses of organizational factors to utilization of evidence based practice, among nurses working in ARRH, Ethiopia, 2019 (n = 671).

**Variables**	**EBP Utilization**			
	**Good N (%)**	**Poor N (%)**	**COR (95%CI)**	**AOR (95%CI)**
Presence of evidence-based guideline at the work area				
Present	274 (40.8%)	135 (20.1%)	3.568 (2.577-4.940)	1.827 (1.249-2.673)*
Absent	95 (14.2%)	167 (24.9%)	1	1
Internet access at the work place				
Yes	237 (35.3%)	117 (17.4%)	2.839 (2.073-3.888)	1.655 (1.119-2.448)*
No	132 (19.7%)	185 (27.6%)	1	1
Computer availability at the work area				
Available	210 (31.3%)	97 (14.5%)	2.791 (2.032-3.834)	1.439 (0.962-2.152)
Not available	159 (23.7%)	205 (30.6%)	1	1
Library with updated references				
Present	194 (28.9%)	111 (16.5%)	1.908 (1.398-2.602)	0.878 (0.583-1.322)
Absent	175 (26.1%)	191 (28.5%)	1	1
Presence of a nurse manager who leads and facilitates EBP utilization				
Present	204 (30.4%)	100 (14.9%)	2.497 (1.822-3.424)	1.359 (0.920 2.008)
Absent	165 (24.6%)	202 (30.1%)	1	1
Presence of enough time at the workplace				
Yes	174 (25.9%)	88 (13.1%)	2.170 (1.573-2.993)	0.851 (0.541-1.339)
No	195 (29.1%)	214 (31.9%)	1	1
Patient overload (patient to nurse ratio)				
≤ 6:1	161 (24.0%)	128 (19.1%)	1.052 (0.774-1.431)	
> 6:1	208 (31.0%)	174 (25.9%)	1	
Work load				
≤39 hours/ week	157 (23.4%)	127 (18.9%)	1.020 (0.750-1.388)	
>39 hours/week	212 (31.6%)	175 (26.1%)	1	

Where: 1 = Reference, ** = p< 0.001, * = p< 0.05

Discussion

In this study, 55% (95% CI: 51.2, 58.9) of nurses had good evidence-based practice utilization. This finding is comparable to the study done in Ethiopia (28, 39), Kenya (27), Nigeria (26) and Zambia (24) (37) where 51.8% and 57.6%, 39.9%, 53.6% and 54% of nurses utilized research driven evidence during care provision respectively.

But, this finding is lower when compared to the study conducted in southern Ethiopia and southwest Ethiopia, where 61.5% (29) and 81.1% (40) of nurses utilized EBP during their patient care respectively. This inconsistency could be from lack of training and knowledge difference. In this study, only 23 % of nurses received EBP training. Again, from those studies it was reported that most of their participants had better knowledge about EBP as a majority, 81.2% (29) and 62.9% (40) of their respondents were familiar with the concept of EBP than this study (34.6%). Hence, knowledgeable nurses are confident and eager to integrate evidences in their practice as compared to their counterparts (28).

This finding is higher than the study done in Ghana, Australia and Iran whereby only 25.3% (25), one-third (21) and 41% (22) of nurses had utilized evidence-based practice respectively. This could be due to difference in knowledge, attitude, training and operational definition from study to study. Additionally, it could associate that the majority of health workers, including nurses in Africa did not learn about EBP in nursing schools (41).

After adjusting for possible confounding factors, it was found that marital status, work experience, knowledge, communication skill, internet access, training and evidence-based guideline availability were significantly associated with EBP utilization.

This study showed that, marital status had significant association with EBP utilization. It was found that nurses who were single in marital status were 66.2% more likely (AOR = 1.662, 95% CI: 1.089, 2.536) to have good EBP utilization than married nurses. This is in contrast with the study of Kenya (42) in which marital status has no relationship with utilization of EBP but analogous with other study (29). The reason could be due to the fact that married nurses have added duties at home, which put them to the extra workload. Due to this, married nurses might not search, appraise and integrate evidences properly.

In this study, work experience was significantly associated with utilization of EBP. Nurses who had worked for less than 5 years and 5-10 years were about 85% (AOR = 1.849, 95% CI: 1.049, 3.257) and 2.2 times (AOR = 2.227, 95% CI: 1.271, 3.900) more likely to have good EBP utilization compared to those who had worked more than 10 years respectively. This finding is consistent with the study from Iran (43) but, reverse to the study from Ethiopia (39) and Norway (20) that stated work experience had no association and having fewer work experience had preventive association to EBP respectively. This discrepancy may be due to differences in study period. But, the possible reason for the above finding could be that those nurses who have ten or more years working experience may not get the opportunity of learning EBP at nursing school since EBP is a new approach in the region and may not update their knowledge as science is dynamic. Additionally, younger nurses are better in valuing EBP and elders were tending to use self-experience (20).

This study revealed that having good knowledge about EBP was 2 times (AOR= 2.044, 95% CI: 1.406-2.972) more likely to have good EBP utilization compared to having poor knowledge about EBP. The possible explanation for this could be knowledge about EBP may increase their appraisal skills and give more confidence in utilizing EBP. Additionally, the large proportion of our respondents were in the younger age group (21-30 years), who might be active in information sharing. This finding is supported by results from Ethiopia (28, 39, 40), Malaysia (44) and US (45) whereby nurses who knew about evidence-based practice were more likely to utilize evidence-based practice than those who did not know.

This study was added a new predictor variable, communication skill, and has got a significant association with EBP utilization. Those nurses who had effective communication with the patient were 2.5 times (AOR= 2.537, 95% CI: 1.744-3.689) more likely to have good EBP utilization as compared to those who had ineffective communication. The reason for this finding might be due to the fact that having effective communication with the patient can prevent the missing and wrong interpretation of patient's important information and creates a trustworthy environment between the two. This may have contributed in integrating patient's values and preferences. Additionally, this might be due to the notion that nurses' communication skill is the key to quality of patient care (38).

Similar to studies conducted in Ethiopia (31) and Iran (22), this study also found out that training about EBP was significantly associated with utilization of EBP. It was observed that nurses who attend EBP training either as part of their curriculum or separately are 3.2 times (AOR= 3.224, 95% CI: 1.957-5.311) more likely to have good EBP utilization compared to those who do not attend it. The reason could be that training may help nurses to be clear more about the steps of EBP. Again, study found that those respondents who had attended EBP training got themselves more comfortable in integrating EBP into their practice (46).

Internet access was also found one of the factors significantly associated with EBP utilization. Nurses who had internet access at work area were 65.5% more (AOR= 1.655, 95% CI: 1.119-2.448) likely to have good EBP utilization as compared to those who hadn't. This finding is in agreement with studies done in Ethiopia (31), Ghana (25), Uganda (47), Norway (20), Malaysia (44) and US (45). This is due to the fact that EBP utilization needs some prerequisites like searching over the internet and easy access to online EBP resources may simplify the use of evidence-based practice (1).

Furthermore, this study found that evidence-based guideline availability in the work area had significant association with evidence-based practice utilization. Nurses who had guidelines at the work were 1.8 times more likely to have good EBP utilization as compared to their counterparts (AOR= 1.827, 95% CI: 1.249-2.673). This finding is consistent with studies done in Ethiopia (28) and USA (48) that states lack of updated guidelines and organizational factors like absence of resources to be accessed in the work area is the hindering factor from utilizing EBP. The reason may possibly be that if evidence-based guidelines are available at work area, nurses could refer them at the bedside and provide scientific based care without delay.

Incorporating one important predictor variable, communication skill, makes this study stronger. But, use of self-administered questionnaire may end up with response bias and as per researchers literature review, this study was done on certain variables. It would have been worthy if role at the hospital and shifting type had been determined.

Conclusions

Even though more than half (55%) of nurses had good utilization, evidence-based practice in this study was found to be low as compared to studies conducted in other parts of Ethiopia. Being singe in marital status, having fewer work experience, good EBP knowledge, effective communication skill, taking EBP training, internet access and having evidence-based guideline at the work area were predictor variables to EBP utilization. Hence, orienting early graduated nurses, building nurses' EBP competencies through either by giving separate EBP training or incorporating EBP as part of the curriculum, availing evidence-based guidelines in the work area and improving hospitals internet access are decisive to improve EBP. Similarly, communication skill was emerging as a predicting variable to EBP utilization. So, nurses need to give attention about it.

Abbreviations

AOR: Adjusted Odds Ratio; ARRH: Amhara Region Referral Hospitals; CI: Confidence Interval; COR: Crude Odds Ratio; EBP: Evidence-Based Practice; ETB: Ethiopian Birr; IQR: Inter Quartile Range

Supporting Information

S1 Text. Questionnaire.

(DOCX)

Data Availability Statement

All relevant data are within the manuscript.

Competing interests

The authors have declared that no competing interests exist.

Funding statement

Amhara Regional Health Bureau paid per diem for data collectors during data collection. Thus, it had no role in study design, data collection and analysis, decision to publish, or preparation of the manuscript.

Authors' contributions

ZBA: Conceived and designed the study, facilitated the data collection, performed data analysis, data interpretation and drafted the manuscript. KGY and MHG participated in designing the study, data analysis and interpretation, and editing the manuscript. All authors read and approved the final manuscript.

Acknowledgment

First, we would like to forward our heartfelt gratitude to Amhara Regional Health Bureau for its financial support and all Referral Hospital Matrons in the region for their valuable study setting information. Secondly, we want to thank all supervisors, data collectors and study participants. Finally, we would like to give our greatest thanks to University of Gondar, School of Nursing for offering the opportunity to carry out this study.

References

1. Melnyk BM, Fineout-Overholt E. Evidence-based practice in nursing & healthcare: A guide to best practice: Lippincott Williams & Wilkins; 2011.
2. Leen B, Bell M, McQuillan P. Evidence-based practice: a practice manual. 2014.
3. Thompson CJ. What does "Best Evidence" mean in evidence-based practice? [Blogpost]. Retrieved from https://nursingeducationexpertcom/what-does-best-evidence-mean-ebp/. 2016, December 20. (Accessed date on 18/02/2019).
4. Thompson CJ. What does "clinical expertise" mean in evidence-based practice? [Blogpost]. Retrieved from https://nursingeducationexpertcom/what-does-clinical-expertise-mean-ebp/. 2016, December 27. (Accessed date on 18/02/2019).
5. Thompson CJ. What does "patient preferences" mean in evidence-based practice? [Blogpost]. Retrieved from https://nursingeducationexpertcom/what-does-patient-preferences-mean-ebp/. 2017, January 10. (Accessed date on 18/02/2019).

6. Virpi Jylhä, Ashlee Oikarainen, Marja-Leena Perälä, Arja Holopainen. Facilitating evidence-based practice in nursing and midwifery in the WHO European Region. 2017. Available at: www.euro.who.int/data/assets/pdffile/0017//WH06EBPreportcomplete.pdf. (Accessed on December 12, 2018).
7. Famutimi Esther Oluwatoyin, FWACN Justina H. Eke, Fowomola BB. Knowledge and Utilization of Evidence-Based Nursing Practice among Nurses of Offa Specialist Hospital, Kwara State. IOSR Journal of Nursing and Health Science (IOSR-JNHS). 2015;4(4):51-62.
8. Branham S, DelloSritto R, Hilliard T. Lost in translation: The acute care nurse practitioners' use of evidence based practice: A qualitative study. Journal of nursing education and practice. 2014;4(6):53.
9. Melnyk BM, Fineout-Overholt E, Gallagher-Ford L, Kaplan L. The state of evidence-based practice in US nurses: critical implications for nurse leaders and educators. Journal of Nursing Administration. 2012;42(9):410-7.
10. Jordan P, Bowers C, Morton D. Barriers to implementing evidence-based practice in a private intensive care unit in the Eastern Cape. Southern African Journal of Critical Care. 2016;32(2):50-4.
11. Kim SC, Stichler JF, Ecoff L, Brown CE, Gallo AM, Davidson JE. Predictors of Evidence-Based Practice Implementation, Job Satisfaction, and Group Cohesion Among Regional Fellowship Program Participants. Worldviews on Evidence-Based Nursing. 2016;13(5):340-8.
12. Jane Vortherms M, Brenda Spoden B, Jill Wilcken B. From evidence to practice: developing an outpatient acuity-based staffing model. Clinical journal of oncology nursing. 2015;19(3):332.
13. Saleh US. Theory guided practice in nursing. Journal of Nursing Research and Practice. 2018;2(1).
14. Muhumuza C, Gomersall JS, Fredrick ME, Atuyambe L, Okiira C, Mukose A, et al. Health care worker hand hygiene in the pediatric special care unit at Mulago National Referral Hospital in Uganda: a best practice implementation project. International journal of evidence-based healthcare. 2015;13(1):19-27.
15. Feyissa GT, Gomersall JS, Robertson-Malt S. Compliance to hand hygiene practice among nurses in Jimma University Specialized Hospital in Ethiopia: a best practice implementation project. JBI database of systematic reviews and implementation reports. 2014;12(1):318-37.

16. Aurang Z MR, Sireeta E NR, Saima Q, et al. Barriers to Evidence Based Nursing Practice in Tertiary Care Hospitals of Peshawar, Pakistan. Biomed J Sci & Tech Res. 2018;8(1).
17. Fairbrother G, Cashin A, Conway MR, Symes MA, Graham I. Evidence based nursing and midwifery practice in a regional Australian healthcare setting: Behaviours, skills and barriers. Collegian (Royal College of Nursing, Australia). 2016;23(1):29-37.
18. Sadeghi-Bazargani H, Tabrizi JS, Azami-Aghdash S. Barriers to evidence-based medicine: a systematic review. Journal of evaluation in clinical practice. 2014;20(6):793-802.
19. Stokke K, Olsen NR, Espehaug B, Nortvedt MW. Evidence based practice beliefs and implementation among nurses: a cross-sectional study. BMC nursing. 2014;13(1):8.
20. Dalheim A, Harthug S, Nilsen RM, Nortvedt MW. Factors influencing the development of evidence-based practice among nurses: a self-report survey. BMC health services research. 2012;12(1):367.
21. Koschel A, Cross M, Haines H, Ervin K, Skinner-Louis D, Carbone D. Research and evidence based practice in a rural Victorian cohort. Australian Journal of Advanced Nursing, The. 2012;30(2):13.
22. Mozafarpour S, Sadeghizadeh A, Kabiri P, Taheri H, Attaei M, Khalighinezhad N. Evidence-based medical practice in developing countries: the case study of Iran. Journal of evaluation in clinical practice. 2011;17(4):651-6.
23. Kabita Paudel, Isabel L. Perceived Barriers To Use of Evidence Based Practices Among Nurses of A Teaching Hospital. IOSR Journal of Nursing and Health Science (IOSR-JNHS). 2018;7(2):29-35.
24. Wamunyima M, Akakandelwa Dr A, Ms K, Wamunyima C. Nurses and Use of Research Information in Clinical Practice: a Case Study of the University Teaching Hospital in Zambia. 2017.
25. Nkrumah I, Atuhaire C, Priebe G, Cumber SN. Barriers for nurses' participation in and utilisation of clinical research in three hospitals within the Kumasi Metropolis, Ghana. The Pan African medical journal. 2018;30.
26. Baiomy S, Khalek EMA. Factors influencing effective implementation of evidence based practice among nurses in assiut city hospitals , Egypt : A comparative Study. IOSR J Nurs Heal Sci. 2015;4:11-9.

27. Mutisya AK, KagureKarani A, Kigondu C. Research utilization among nurses at a teaching hospital in Kenya. Journal of caring sciences. 2015;4(2):95.
28. Dereje B, Hailu E, Beharu M. Evidence-based Practice Utilization and Associated Factors among Nurses Working in Public Hospitals of Jimma Zone Southwest Ethiopia: A Cross Sectional Study. Adv Pharmacoepidemiol Drug Saf. 2019;7:321.
29. Tadesse B, Eyoel AD, Teshome HW. Assessment of Nurses Knowledge and Utilization of Evidence based Practice and its Associated Factors in Selected Hospitals of Southern Ethiopia. Adv Practice Nurs. 2018;3(2).
30. Gautham M, Berhanu D, Umar N, Ghosh A, Elias N, Spicer N, et al. Panel discussion: The challenges of translating evidence into policy and practice for maternal and newborn health in Ethiopia, Nigeria and India. BMC health services research. 2014;14(2):O7.
31. Beshir MA, Woreta SA, Kebede M. Evidence-based practice among health professionals in hospitals of Northwest Ethiopia: a cross-sectional study. International journal of evidence-based healthcare. 2017;15(4):161-70.
32. Dessie G, Jara D, Alem G, Mulugeta H, Zewedu T. Implementation of Evidence-Based Medicine and Associated Factors among Health Care Providers Working in Public Hospitals, Northwest Ethiopia. Ethiopian Public Health Association (EPHA) 29th Annual Conference. February 26 - 28, 2018, Addis Ababa. Available at https://etphaorg/conference/indexphp/29thConference/29thConference/paper/downloadSuppFile/1044/342. (Accessed on December 12, 2018).
33. Health FDRoEMo. Health Sector Development Program IV, 2010/11—2014/15. Ministry of Health Addis Ababa; 2010.
34. Rice K, Hwang J, Abrefa-Gyan T, Powell K. Evidence-based practice questionnaire: a confirmatory factor analysis in a social work sample. Advances in Social Work. 2010;11(2):158-73.
35. Upton P, Scurlock-Evans L, Upton D. Development of the student evidence-based practice questionnaire (S-EBPQ). Nurse education today. 2016;37:38-44.
36. Ruzafa-Martinez M, Lopez-Iborra L, Moreno-Casbas T, Madrigal-Torres M. Development and validation of the competence in evidence based practice questionnaire (EBP-COQ) among nursing students. BMC medical education. 2013;13(1):19.

37. Yeganeh MR, Pouralizadeh M, Ebadi A. Psychometric Evaluation of Iranian Version of the Evidence-Based Practice Questionnaire (EBPQ): A Methodological Study. Iranian Red Crescent Medical Journal. 2017;19(11).
38. Ayman M, Ahmad E, Aboshaiqah B, Imad N, Wisam M, Salim R, et al. Patients' satisfaction about nurses' competency in practicing communication skills. Life Science Journal. 2014;11(3):339-34.
39. Hadgu G, Almaz S, Tsehay S. Assessment of nurses' perceptions and barriers on evidence based Practice in Tikur Anbessa Specialized Hospital Addis Ababa Ethiopia. Am J Nurs Sci. 2015;4(3):73-83.
40. Hoyiso D, Arega A, Markos T. Evidence based nursing practice and associated factors among nurses working in Jimma zone public hospitals, Southwest Ethiopia. International Journal of Nursing and Midwifery. 2018;10(5):47-53.
41. Omar Abdulwadud, Sailas Nyareza, Floyd Malasha, Mthoniswa JP. Relevance and barriers to evidence-based practice in Zambian Defense Force Medical Services: A qualitative analysis of roundtable group discussion by Military Health Personnel. ARC Journal of Nursing and Healthcare (AJNH). 2017;3(1):1-11.
42. Barako TD, Chege M, Wakasiaka S, Omondi L. Factors influencing application of evidence-based practice among nurses. African Journal of Midwifery and Women's Health. 2012;6(2):71-7.
43. Heydari A, Mazlom SR, Ranjbar H, Scurlock-Evans L. A study of Iranian nurses' and midwives' knowledge, attitudes, and implementation of evidence-based practice: The time for change has arrived. Worldviews on Evidence-Based Nursing. 2014;11(5):325-31.
44. Lai NM, Teng CL, Lee ML. The place and barriers of evidence based practice: knowledge and perceptions of medical, nursing and allied health practitioners in malaysia. BMC research notes. 2010;3(1):279.
45. Tacia L, Biskupski K, Pheley A, Lehto RH. Identifying barriers to evidence-based practice adoption: A focus group study. Clinical Nursing Studies. 2015;3(2):90-6.
46. Majid S, Foo S, Luyt B, Zhang X, Theng Y-L, Chang Y-K, et al. Adopting evidence-based practice in clinical decision making: nurses' perceptions, knowledge, and barriers. Journal of the Medical Library Association: JMLA. 2011;99(3):229.

47. Boer K. barriers to evidence-based practice in doctors and nurses in Rwanda and Uganda: no 02. Tropical Medicine & International Health. 2012;17:69.
48. Boström A-M, Rudman A, Ehrenberg A, Gustavsson JP, Wallin L. Factors associated with evidence-based practice among registered nurses in Sweden: a national cross-sectional study. BMC health services research. 2013;13(1):165.

Click here to access/download
Supporting Information
Questionnaires.doc
