# Supplementary material for: Evidence-based practice utilization and associated factors among nurses working in Amhara Region Referral Hospitals, Ethiopia
Source: PLoS One. 2021 Mar 19;16(3):e0248834. doi: 10.1371/journal.pone.0248834 (PMC7978364; doi:10.1371/journal.pone.0248834)
Supplement: Attachment — Submitted filename: Response to Reviewers.doc [file pone.0248834.s004.doc]

**Dear editors;**

Greetings;

Thank you very much for your valuable suggestions and concerns! All are very constructive.

The detailed response for all suggestions and concerns are summarized in the table below!

Thanking you again!!

| **Reviewer/editors Number** | **Original comments of the reviewer:** | **Reply by the author(s)** | **Changes done on page number and line number** |
| --- | --- | --- | --- |
| **Reviewr#1:** |  |  |  |
| 1. | This is a highly simplistic description of what is EBP. EBP is about much more than this, and descriptions of theoretical frameworks, facilitators, and barriers, systematic reviews/clinical guidelines at a minimum would be expected. Use of the term "best evidence" is also non-standard and confusing. Use of a blog post as a primary reference is inappropriate. Please address all three points above. | Accepted and corrected! | See in the track change document on page 3, line number 49 up to 57, 64, and 71. |
| 2. | Again, an inappropriate citation | Accepted and corrected! | See in the track change document on page 3, line number 64. |
| 3. | The English expression here and earlier parts of the introduction is unclear and needs re-working | Accepted and reworked! | See in the track change document on page 3, line number 67 to 71. |
| 4. | Again, inappropriate citation | Accepted and corrected! | See in the track change document on page 3, line number 71. |
| 5. | The meaning of this sentence is not clear | Accepted and made clear! | See in the track change document on page 3, line number 72 to 74. |
| 6. | Apart from the first and last sentences, this paragraph is better than the preceding two (ie it uses appropriate references and is clearly written). |  | See in the track change document on page 3, line number 79. |
| 7. | It is not clear what these numbers mean | Those numbers refer to the proportions of nurses' evidence-based practice utilization to the mentioned countries. | See in the track change document on page 4, line number 88 and 89. |
| 8. | Again, not clear what these numbers mean | Again those numbers refer to the proportions of nurses' evidence-based practice utilization to the mentioned countries. | See in the track change document on page 4, line number 90 to 92. |
| 9. | All of these sentences need to be re-worked as they are not clear | Accepted and rewritten. | See in the track change document on page 4, line number 93 to 100. |
| 10. | These study objectives have not been properly described in text | Accepted and well described! | See in the track change document on page 5, line number 114 to 122 |
| 11. | As per many sections of the text, this needs rewriting to improve the English expression. | Accepted and modified! | See in the track change document on page 5, line number 140 to 150. |
| 12. | As this is the main outcome of the study, there should be greater clarity about how it was measured, where was the tool derived from, what are its psychometric properties (if known). | We already tried to clearly write the tools used to measure the outcome and independent variables by including the points you raised such as the psychometric properties in other studies and our study. | See in the track change document on page 7, line number 153, 165 up to 169 & 172 up to 178. |
| 13. | What's the rationale for this cut-off? Need a much greater amount of information to ensure that this measure is valid and reliable | Regarding the cut-off point, we already delineated in the document. As evidence shows, When outliers encountered, the median together with the inter-quartile range is the better choice of summary measure instead of mean with a standard deviation. | See in the track change document on page 7, line number 174 to 178. |
| 14. | As per earlier commentary, it is necessary to identify the validity/reliability of this tool and its psychometric properties for all the independent variables as well as the dependent variables | Concerning the tool, we already tried to describe in the document. | See in the track change document on page 7, line number 165 to 169. |
| **Editors** |  |  |  |
| 1. | We note that you have included the questionnaires used in the study as Supporting Information. Please ensure that you have permissions to publish these scales with a CC BY license. We would suggest that you remove any questions that you do not have permission to publish and include a reference instead. For more detail please see here: <https://journals.plos.org/plosone/s/licenses-and-copyright>. | Based on your suggestion we removed the questionnaire section from the document and included the minimal data set. | See in the track change document on page 20, line number 393 to 397. |
| 2. | Please upload a copy of Figure 1, to which you refer in your text on page 13. If the figure is no longer to be included as part of the submission please remove all reference to it within the text. | Accepted and removed | See in the track change document on page 6, line number 150. |
| 3. | We note you have included a table to which you do not refer in the text of your manuscript. Please ensure that you refer to Table 1 in your text; if accepted, production will need this reference to link the reader to the Table. | We really admire your comment and we accept and correct the technical error that we mad. | See in the track change document on page 5, line number 131. |
| 4. | Inappropriate referencing from blog posts | Accepted and corrected! | See in the track change document on page 3, line number 57, 64, and 71. |
| 5. | Poor English expression. A number of incidences are marked up, but the whole manuscript requires editing for English expression. | Accepted and reworked! | The whole document. |
| 6. | Lack of clarity about the tools that were used to measure the outcome and the independent variables. How were they derived? | We already tried to clearly write the tools used to measure the outcome and independent variables by including the points you raised such as the psychometric properties in other studies and in our study, justifications for the use of the cut-point to say good and bad EBP. | See in the track change document on page 7, line number 153, 164 up to 169, 172 up to 178, 179 up to 187, 188 up to 194 and 195 up to 202. |
| 6.1. | What are the psychometric properties? | Previously stated | See in the track change document on page 7, line number 165 to 169. |
| 6.2. | Have they been validated in other prior studies? | Already mentioned | See in the track change document on page 7, line number 164, and 167 to 169. |
| 6.3. | What is the justification for the use of cut-off between what is good and bad EBP? | To categorize as good and poor EBP: first, we test the data set for normality using the Shapiro–Wilk test and got non-normal data due to outliers.  Then, when outliers encountered, the median together with the inter-quartile range is the better choice of summary measure instead of mean with a standard deviation.  Last, we categorized participants who scored equal and above the cutoff point (Median score) as having “good EBP” otherwise “poor EBP”. | See in the track change document on page 7, line number 174 to 178 and on page 8, line number 182 to 184, 191 to 192 and 198 up to 200. |
|  | **Again we would like to say thank you for all given comments and your time!** | |  |
