# Supplementary material for: Evidence-based practice utilization and associated factors among nurses working in Amhara Region Referral Hospitals, Ethiopia
Source: PLoS One. 2021 Mar 19;16(3):e0248834. doi: 10.1371/journal.pone.0248834 (PMC7978364; doi:10.1371/journal.pone.0248834)
Supplement: Attachment — Submitted filename: review on PlOS ONE paper.docx [file pone.0248834.s005.docx]

**Abstract**

‘’ Despite having the above positive effects on patient outcomes, nurses do not onsistently utilize evidence-based practice.’’ So what is need of research if you have already known that nurses are not utilizing evidence-based practice? You have to put here the clear gap of your research area.

**Introduction**

I haven’t seen the clear gap of your research area. So it will be better if you write the gap or the main problem related with your research area. Also consider these issues in your introduction part; major factors that influence the problem, solutions that have been tried in the past, how well the solutions worked and why further research is needed.

**Method part**

You said ‘’Nurses who had work experience of 06 months and above were included in the study.’’ Why you excluded those nurses who had work experience of less than 06 months? I think nurses with low work experience may utilize EBP. What is the scientific reason behind that How do you know they don’t utilize evidence based practice??

Sampling technique is not clear. Describe it well about your sampling technique

Data collection procedure is not clear, describe it well

# Description about the measurement of your study variable should be written under the sub heading of ‘’Instrument and measurement’’ separately.

# What is EBP in your study? Operationalize it

# Result

# What is the base for you to categorize Nurses monthly salary (ETB) categorization? It needs scientific reason.

**Evidence-based practice utilization**

**You said ‘**To measure nurses’ EBP utilization, six questions were asked. Each question had a 5-point Likert scale’’…..in the method part, what are those questions? It will be better if you present descriptions of the EBP utilization on table with frequency (number and percentage) of each six items.

**Recommendation**

I didn’t see recommendation on you study based on your study findings. Describe it briefly and it has to consider *for whom, by whom and what will be done, and it should be feasible*
