# Supplementary material for: Evidence-based practice utilization and associated factors among nurses working in Amhara Region Referral Hospitals, Ethiopia
Source: PLoS One. 2021 Mar 19;16(3):e0248834. doi: 10.1371/journal.pone.0248834 (PMC7978364; doi:10.1371/journal.pone.0248834)
Supplement: Attachment — Submitted filename: Response to Reviewers.doc [file pone.0248834.s007.doc]

Date: November 21, 2020

**Rebuttal letter**

**Dear Editor,**

Thank you for allowing us the opportunity to submit a revised draft of the manuscript “Evidence-Based Practice Utilization and Associated Factors among Nurses working in Amhara Region Referral Hospitals, Ethiopia” for publication in the Journal of PLOS ONE. We appreciate the efforts that you and the reviewers devoted to providing feedback on our manuscript. All are valuable and insightful comments that led to possible improvements in the current version. We have carefully considered all suggestions and concerns and tried our best to address every one of them.

Please see below, in blue, for a point-by-point response to the reviewers. All page numbers refer to the revised manuscript file with tracked changes. All modifications in the manuscript have been highlighted in red.

Best regards,

Zewdu Bishaw

[Zest7@yahoo.com](mailto:Zest7@yahoo.com)

Head, Department of Nursing at Bahir Dar Health Science College

Thank you for submitting your manuscript to PLOS ONE. After careful consideration, we feel that it has merit but does not fully meet PLOS ONE’s publication criteria as it currently stands. Therefore, we invite you to submit a revised version of the manuscript that addresses the points raised during the review process.


Please submit your revised manuscript by Nov 22 2020 11:59PM. If you will need more time than this to complete your revisions, please reply to this message or contact the journal office at plosone@plos.org. When you're ready to submit your revision, log on to https://www.editorialmanager.com/pone/ and select the 'Submissions Needing Revision' folder to locate your manuscript file.

Please include the following items when submitting your revised manuscript:

- A rebuttal letter that responds to each point raised by the academic editor and reviewer(s). You should upload this letter as a separate file labeled 'Response to Reviewers'.
- A marked-up copy of your manuscript that highlights changes made to the original version. You should upload this as a separate file labeled 'Revised Manuscript with Track Changes'.
- An unmarked version of your revised paper without tracked changes. You should upload this as a separate file labeled 'Manuscript'.

If you would like to make changes to your financial disclosure, please include your updated statement in your cover letter. Guidelines for resubmitting your figure files are available below the reviewer comments at the end of this letter.

If applicable, we recommend that you deposit your laboratory protocols in protocols.io to enhance the reproducibility of your results. Protocols.io assigns your protocol its own identifier (DOI) so that it can be cited independently in the future. For instructions see: [http://journals.plos.org/plosone/s/submission-guidelines#loc-laboratory-protocols](http://journals.plos.org/plosone/s/submission-guidelines#loc-laboratory-protocols)

**Authors response: we i**ncluded the DOI link in the Methods section of the manuscript

Additional Editor Comments (if provided):

Please see comments from two reviewers below. All comments need to be addressed. Additionally please ensure that the manuscript complies with the STROBE checklist for cross-sectional studies and upload a completed STROBE checklist.

[Note: HTML markup is below. Please do not edit.]

**Reviewers' comments:

Reviewer's Responses to Questions**

**Comments to the Author**

1. If the authors have adequately addressed your comments raised in a previous round of review and you feel that this manuscript is now acceptable for publication, you may indicate that here to bypass the “Comments to the Author” section, enter your conflict of interest statement in the “Confidential to Editor” section, and submit your "Accept" recommendation.

Reviewer #1: (No Response)

Reviewer #2: (No Response)

**Author/s response:** Thank you for your previous comments. We have gone through your comments carefully and tried our best to address them one by one. We hope the manuscript has been improved accordingly.

2. Is the manuscript technically sound, and do the data support the conclusions?

The manuscript must describe a technically sound piece of scientific research with data that supports the conclusions. Experiments must have been conducted rigorously, with appropriate controls, replication, and sample sizes. The conclusions must be drawn appropriately based on the data presented.

Reviewer #1: Yes

Reviewer #2: Partly

**Author/s response**: We truly appreciate the reviewer’s concerns, and we recognize that it would improve the revised version of the manuscript. As it stands, the conclusion was made based on our findings.

3. Has the statistical analysis been performed appropriately and rigorously?

Reviewer #1: Yes

Reviewer #2: No

**Author/s response:** We appreciate the concern you raised and we also agree that multiple linear regression is a suitable model. The proposed model of analysis at the proposal stage was also linear regression. However, the data does not fulfill all the assumptions for multiple linear regressions like normality, linearity, and homoscedasticity. Therefore, we were forced to dichotomize and conduct binary logistic regression. Though less favored, logistic regression is commonly used in many articles and we hope ours will have a lot to tell for the scientific community.

4. Have the authors made all data underlying the findings in their manuscript fully available?

The [PLOS Data policy](http://www.plosone.org/static/policies.action#sharing) requires authors to make all data underlying the findings described in their manuscript fully available without restriction, with rare exception (please refer to the Data Availability Statement in the manuscript PDF file). The data should be provided as part of the manuscript or its supporting information, or deposited to a public repository. For example, in addition to summary statistics, the data points behind means, medians and variance measures should be available. If there are restrictions on publicly sharing data—e.g. participant privacy or use of data from a third party—those must be specified.

Reviewer #1: Yes

Reviewer #2: Yes

5. Is the manuscript presented in an intelligible fashion and written in standard English?

PLOS ONE does not copyedit accepted manuscripts, so the language in submitted articles must be clear, correct, and unambiguous. Any typographical or grammatical errors should be corrected at revision, so please note any specific errors here.

Reviewer #1: Yes

Reviewer #2: No

**Author/s response**: We went through the entire manuscript to eliminate grammatical mistakes.

6. Review Comments to the Author

Please use the space provided to explain your answers to the questions above. You may also include additional comments for the author, including concerns about dual publication, research ethics, or publication ethics. (Please upload your review as an attachment if it exceeds 20,000 characters)

Reviewer #1: (No Response)

Reviewer #2: Thank-you for the opportunity to review R1 of this manuscript. While it does have its strengths, including a high response rate and a large sample size, there are some issues that need to be addressed. My main criticism reflects my comment from the first version of the manuscript in relation to the use of cut-off scores to dichotomise a variable that could be considered continuous (eg EBP utilisation on a range of 6-30) into eg low and high, or poor and good. This analysis has two implications: with a cut-off of the median 16, a score of 15 is considered equivalent to a score of 10 and 5. A lot of variation in the data is consequently lost. Second, if the cut-off is 16 and above, then of course a little more than 50% of the responses are going to fall in this category. So, the main finding that 55% of nurses had good EBP utilisation is completely meaningless.
I would recommend using multiple regression on the total EBP utilisation score (6-30) instead. And this suggestion also applies to the predictors Knowledge, Attitude and Communication Skill. They could all be added as continuous predictors in a multiple regression.
There are a range of other criticisms of the paper included on the PDF of the manuscript. I believe that all should be addressed including:
• Structure of the tables (there are too many tables)
• Numerous instances of poor English expression
• Remove the general objectives
• Clarifying why some of the variables don’t have adjusted odds ratios given (eg see comment on page 15)

Author response: Thank you for these concerns. We removed Tables 3 and 4 and state findings in narrative form, the English expression was reworked, the general objective was removed, etc (For more, see detailed response under each point).

7. PLOS authors have the option to publish the peer review history of their article ([what does this mean?](https://journals.plos.org/plosone/s/editorial-and-peer-review-process#loc-peer-review-history)). If published, this will include your full peer review and any attached files.


If you choose “no”, your identity will remain anonymous but your review may still be made public.


**Do you want your identity to be public for this peer review?** For information about this choice, including consent withdrawal, please see our [Privacy Policy](https://www.plos.org/privacy-policy).

Reviewer #1: No

Reviewer #2: **Yes:** Tim Schultz

**Reviewers' Comments to the Authors:**

**Reviewer #1**

1. Abstract: ‘’ Despite having the above positive effects on patient outcomes, nurses do not consistently utilize evidence-based practice.’’ So what is need of research if you have already known that nurses are not utilizing evidence-based practice? You have to put here the clear gap of your research area

Author/s response: Thank you for pointing this out. We accepted and reworked! See in the track change document on page 2, line number 21 up to 27.

2. Introduction: I haven’t seen the clear gap of your research area. So it will be better if you write the gap or the main problem related with your research area. Also consider these issues in your introduction part; major factors that influence the problem, solutions that have been tried in the past, how well the solutions worked and why further research is needed

Author/s response: We agree with these and have incorporated your valuable comments. See in the track change document on page 4, line number 80 up to 86 and 93 up to 106, and on page 5, line number 107 up to 115.

3. **Method part**
   3.1. You said ‘’Nurses who had work experience of 06 months and above were included in the study.’’ Why you excluded those nurses who had work experience of less than 06 months? I think nurses with low work experience may utilize EBP. What is the scientific reason behind that How do you know they don’t utilize evidence based practice??

**Author/s response:** We appreciate the reviewer’s comments. But according to labour law of Ethiopia, any newly appointed civil servant, including we nurses, will undergo a probation period. This period lasts for six months. During this time his/her department head will closely observe, monitor, and supervise him/her but no his/her independent performances will be evaluated. Yes, this period is allocated to test a person’s suitability for a post in which he/she is expected to be assigned. No newly appointed employee let to perform independently but under supervision. Since no independent performance evaluation was made, we excluded those who had work experience of fewer than six months. For detail see at <https://chilot.me/wp-content/uploads/2017/10/federal-civil-servants-proclamation.pdf>.

3.2. Sampling technique is not clear. Describe it well about your sampling technique

Author/s response: Accepted and made clear! See the track change document on page 7, line number 167-8.

3.3. Data collection procedure is not clear, describe it well

Author/s response: Accepted and reworked. See in the track change document on page 7, line number 163 to 168.

3.4. Description about the measurement of your study variable should be written under the sub heading of ‘’Instrument and measurement’’ separately.

Author/s response: We rewrite as suggested. See the track change document on page 7, line number 174.

3.5. What is EBP in your study? Operationalize it

Author/s response: You have raised an important point here. However, we already defined in the introduction part. Operationalizing it might not add any value, except wordiness. See in the track change document on page 2, line number 50 up to 54.

# 4. Result: What is the base for you to categorize Nurses monthly salary (ETB) categorization? It needs scientific reason.

# Author/s response: Thank you for your genuine review. We categorized based on quartile classification; See in the track change document on page 10, line number 257 up to 259.

5. **Evidence-based practice utilization: You said ‘**To measure nurses’ EBP utilization, six questions were asked. Each question had a 5-point Likert scale’’…..in the method part, what are those questions? It will be better if you present descriptions of the EBP utilization on table with frequency (number and percentage) of each six items..

**Author/s response:** Thank you for pointing this out. We agree with this comment. Therefore, we have presented those questions in table form. See in the track change document on page 14, line number 293 up to 299.

6. **Recommendation:** I didn’t see recommendation on you study based on your study findings. Describe it briefly and it has to consider *for whom, by whom and what will be done, and it should be feasible*

**Author/s response:** Accepted and reworked! See in the track change document on page 22, line number 430 up to 444.

**Reviewer#2**

**General comments**

Thank-you for the opportunity to review R1 of this manuscript. While it does have its strengths, including a high response rate and a large sample size, there are some issues that need to be addressed. My main criticism reflects my comment from the first version of the manuscript in relation to the use of cut-off scores to dichotomise a variable that could be considered continuous (eg EBP utilisation on a range of 6-30) into eg low and high, or poor and good. This analysis has two implications: with a cut-off of the median 16, a score of 15 is considered equivalent to a score of 10 and 5. A lot of variation in the data is consequently lost. Second, if the cut-off is 16 and above, then of course a little more than 50% of the responses are going to fall in this category. So, the main finding that 55% of nurses had good EBP utilisation is completely meaningless. I would recommend using multiple regression on the total EBP utilisation score (6-30) instead. And this suggestion also applies to the predictors Knowledge, Attitude and Communication Skill. They could all be added as continuous predictors in a multiple regression.

**Author/s response:** We appreciate the concern you raised and we also agree that multiple linear regression is a suitable model. The proposed model of analysis at the proposal stage was also linear regression. However, the data does not fulfill all the assumptions for multiple linear regressions like, normality, linearity, and homoscedasticity. Therefore, we were forced to dichotomize and conduct binary logistic regression. Though less favored, logistic regression is commonly used in many articles and we hope ours will have a lot to tell for the scientific community.

Other concerns are reworked in the specific comments section below.

**Specific Comments**

**There are a range of other criticisms of the paper included on the PDF of the manuscript. I believe that all should be addressed including:**

1. Structure of the tables (there are too many tables)

**Author/s response:** We have agreed with your comment and reduced tables posted regarding predictor variables. See in the track change document on page 12, line number 271 to 274, and on page 13, line number 283 to 286.

2. Remove the general objectives. These are just repetitions of each other., Suggest delete the general objective

Author/s response: Thank you. The authors removed the general objective as per the comment. See in the track change document on page 6, line number 140 up to 143.

3. It is unusual not to mention systematic reviews in the context of evidence-based practice **Author/s response:** Thank you for your kind review. We agree with you.
4. Not a sentence.

**Author/s response:** Thank you. We have revised the sentence. See in the track change document on page 3, line number 68.

5. Not clear why it is a surprise

**Author/s response:** Agree & replaced with the word “honestly”. See in the track change document on page 3, line number 75.

6. ~~whether good or evil~~

**Author/s response:** Agree, we have removed “whether good or evil” from the sentence. See in the track change document on page 4, line number 90.

7. rephrase – unclear

Author/s response: Accepted and corrected! See the track change document on page 4, line number 91.

8. Rephrase - poorly expressed.

Author/s response: Accepted and rephrased. See in the track change document on page 4, line number 93 to 95.

9. Rephrase - "rather it relies on experience"

Author/s response: Accepted and corrected! See in the track change document on page 5, line number 107.

10. be more consistent throughout about the order of presenting and discussing these measures

Author/s response: We are grateful for this comment as it improves the logical flow of our work. See in the track change document on page 8, line number 177 up to 179.

11. I don't believe that these 'associated factors' are properly introduced below

Author/s response: We agree with the reviewer and have made the revision. See in the track change document on page 12, line number 264 up to 271, and on page 13, line number 276 up to 283.

12. I don't like the fact that a person scoring 15 is treated as the same as a person who scored 10 or 5. I'm not sure why the outcome had to be dichotomized. Why wasn't multiple regression considered (or alternatively, why did binary logistic regression HAVE to be conducted). By dichotomizing the data, a large amount of nuance is lost

**Author/s response:** We appreciate the concern you raised and we also agree that multiple linear regression is a suitable model. The proposed model of analysis at the proposal stage was also linear regression. However, the data does not fulfill all the assumptions for multiple linear regressions like, normality, linearity, and homoscedasticity. Therefore, we were forced to dichotomize and conduct binary logistic regression. Though less favored, logistic regression is commonly used in many articles and we hope ours will have a lot to tell for the scientific community.

13. Is there an approval number?

Author/s response: Indeed. See in the track change document on page 10, line number 242.

14. move up on row

Author/s response: Accepted and corrected. See the track change document on page 11, line number 261.

15. move up

Author/s response: Accepted and corrected. See the track change document on page 11, line number 261.

16. move up one row

Author/s response: Accepted and corrected. See the track change document on page 11, line number 261.

17. given that you used the median as the cut-off, it is not surprising that you have ~49% as 'poor'

Author/s response: We really appreciate the suggestion but we considered the nature of the data set for normality.

18. based on my understanding, these 6 items measuring EBP utilization were measured by a 5 point Likert scale, not a dichotomous yes/.no

Author/s response: Thank you for your kind review. However, we felt sorry for the confusion. These items/variables are individual-level factors. For more understanding, we presented those 6 items measuring EBP utilization using the Likert scale in table form. See in the track change document on page 14, line number 298 up to 299.

19. Are these the "associated factors" mentioned in the methods?

Author/s response: Of course yes. Since, our associated factors were socio-demographic, individual, and organizational related factors. We changed “organizational characteristics” with “organizational related factors”. Now, these factors were properly described in the methods part and factor part. See in the track change document on page 8, line number 179, and on page 13 line number 276 to 283.

20. see earlier comment - better to present under separate columns for high and poor utilization

Author/s response: the purpose of this table was to point out the frequency of organizational-level factors not for the association. To see the association of these factors, we presented in table 6. See in the track change document on pages 17 and 18, line number 331 to 333.

21. I'm assuming this is a sub-total derived from the 6 questions summarized in Table 3, so this should be moved closer to Table 3?

Author/s response: we say sorry for the misunderstandings we made. The removed table, table 3, was to show the frequency/percentage of individual-level factors, not for EBP utilization. Now we are confident it will be clear for you. Again, we presented the sub-total utilization questions in the table. See in the track change document on page 12, line number 272 to 274, and page 14, line number 298 to 299.

22. I'm assuming that AOR are presented here because the P value for this potential predictor was <0.20 (if so, it would be helpful to explain that somewhere; if not please explain)

Author/s response: Thank you for raising this point. However, already mentioned in the statistical analysis part. See the track change document on page 10, line number 234.

23. Clarifying why some of the variables don’t have adjusted odds ratios given (eg see comment on page 15)

Author/s response: Thank you for the question. That is because those variables having p-value > 0.20 and CI includes 1 in the bi-variable logistic regression model were not entered into the multi-variable logistics model.

24. I am not familiar with all of these studies, but, as you will be well aware, if you use a cut-off around the median to designate what constitutes good and bad, or high and low, then of course, about 50% will be in each group...so I find this main finding (55%) meaningless

Author/s response: While we appreciate the reviewer’s feedback, we respectfully disagree. We think this study makes a valuable contribution to the nursing discipline because it identifies certain facilitating and preventing factors to the utilization of EBP in clinical setup, fills information gap in the study area and other scientific papers follow such a method of measurement when extreme values face.

25. Out of interest, did this relationship still exist if age is added to the final model? It seems likely it is a proxy/surrogate measure for age, and there was a clear increase in the odds of EBP utilization as age decreased...

Author/s response: Yes it seems like a surrogate measure, but what we want to assure is that we put the OR that we got from the final model. See in the track change document on page 19, line number 356 to 358.

26. It is confusing to use % and times increases in the same sentence

Author/s response: Thank you very much for your nice notice. Accepted and corrected accordingly. See the track change document on page 19, line number 364.

**Again we would like to say thank you for all the given comments and your time!**
