# Supplementary material for: Evidence-based practice utilization and associated factors among nurses working in Amhara Region Referral Hospitals, Ethiopia
Source: PLoS One. 2021 Mar 19;16(3):e0248834. doi: 10.1371/journal.pone.0248834 (PMC7978364; doi:10.1371/journal.pone.0248834)
Supplement: Attachment — Submitted filename: Response to Reviewers.doc [file pone.0248834.s008.doc]

Date: February 28, 2021

**Rebuttal letter**

**Dear Editor,**

Thank you for allowing us the opportunity to submit a revised draft of the manuscript “Evidence-Based Practice Utilization and Associated Factors among Nurses working in Amhara Region Referral Hospitals, Ethiopia” for publication in the Journal of PLOS ONE. We appreciate the efforts that you and the reviewers devoted to providing feedback on our manuscript. All are valuable and insightful comments that led to possible improvements in the current version. We have carefully considered all suggestions and concerns and tried our best to address every one of them.

Please see below, in blue, for a point-by-point response to the reviewers. All page numbers refer to the revised manuscript file with tracked changes. All modifications in the manuscript have been highlighted in red.

Best regards,

Zewdu Bishaw

[Zest7@yahoo.com](mailto:Zest7@yahoo.com)

Head, Department of Nursing at Bahir Dar Health Science College

Thank you for submitting your manuscript to PLOS ONE. After careful consideration, we feel that it has merit but does not fully meet PLOS ONE’s publication criteria as it currently stands. Therefore, we invite you to submit a revised version of the manuscript that addresses the points raised during the review process.

Please submit your revised manuscript by 3 March 2021. If you will need more time than this to complete your revisions, please reply to this message or contact the journal office at plosone@plos.org. When you're ready to submit your revision, log on to https://www.editorialmanager.com/pone/ and select the 'Submissions Needing Revision' folder to locate your manuscript file.

Please include the following items when submitting your revised manuscript:

- A rebuttal letter that responds to each point raised by the academic editor and reviewer(s). You should upload this letter as a separate file labeled 'Response to Reviewers'.
- A marked-up copy of your manuscript that highlights changes made to the original version. You should upload this as a separate file labeled 'Revised Manuscript with Track Changes'.
- An unmarked version of your revised paper without tracked changes. You should upload this as a separate file labeled 'Manuscript'.

If you would like to make changes to your financial disclosure, please include your updated statement in your cover letter. Guidelines for resubmitting your figure files are available below the reviewer comments at the end of this letter.

If applicable, we recommend that you deposit your laboratory protocols in protocols.io to enhance the reproducibility of your results. Protocols.io assigns your protocol its own identifier (DOI) so that it can be cited independently in the future. For instructions see: [http://journals.plos.org/plosone/s/submission-guidelines#loc-laboratory-protocols](http://journals.plos.org/plosone/s/submission-guidelines#loc-laboratory-protocols)

We look forward to receiving your revised manuscript.

Kind regards,

Sharon Mary Brownie
Academic Editor
PLOS ONE

**Authors’ response: we i**ncluded the DOI link in the Methods section of the manuscript

**Editor Comments** 
The manuscript is much improved, however, the need for some minor revisions remain. Please address the identified issues.

Reviewers' comments:

Reviewer's Responses to Questions

**Comments to the Author**

1. If the authors have adequately addressed your comments raised in a previous round of review and you feel that this manuscript is now acceptable for publication, you may indicate that here to bypass the “Comments to the Author” section, enter your conflict of interest statement in the “Confidential to Editor” section, and submit your "Accept" recommendation.

Reviewer #1: All comments have been addressed

Reviewer #3: All comments have been addressed

2. Is the manuscript technically sound, and do the data support the conclusions?

The manuscript must describe a technically sound piece of scientific research with data that supports the conclusions. Experiments must have been conducted rigorously, with appropriate controls, replication, and sample sizes. The conclusions must be drawn appropriately based on the data presented.

Reviewer #1: Yes

Reviewer #3: Yes

3. Has the statistical analysis been performed appropriately and rigorously?

Reviewer #1: Yes

Reviewer #3: Yes

4. Have the authors made all data underlying the findings in their manuscript fully available?

The [PLOS Data policy](http://www.plosone.org/static/policies.action#sharing) requires authors to make all data underlying the findings described in their manuscript fully available without restriction, with rare exception (please refer to the Data Availability Statement in the manuscript PDF file). The data should be provided as part of the manuscript or its supporting information, or deposited to a public repository. For example, in addition to summary statistics, the data points behind means, medians and variance measures should be available. If there are restrictions on publicly sharing data—e.g. participant privacy or use of data from a third party—those must be specified.

Reviewer #1: Yes

Reviewer #3: Yes

Reviewer #1: Yes

Reviewer #3: Yes

5. Is the manuscript presented in an intelligible fashion and written in standard English?

PLOS ONE does not copyedit accepted manuscripts, so the language in submitted articles must be clear, correct, and unambiguous. Any typographical or grammatical errors should be corrected at revision, so please note any specific errors here.

Reviewer #1: Yes

Reviewer #3: Yes

6. Review Comments to the Author

Please use the space provided to explain your answers to the questions above. You may also include additional comments for the author, including concerns about dual publication, research ethics, or publication ethics. (Please upload your review as an attachment if it exceeds 20,000 characters)

Reviewer #1: (No Response)

Reviewer #3: I will like to thank the researchers for addressing the comments of the reviewers. However, there remains some discretionary but important comments to address that will strengthen the manuscript.

Please see comments below:

1) On page 6, under the heading Study design, setting, and participants, the authors need to rephrase the sentence "During the study period, there were a total of 458, 390, 254, 140 and 223". The authors should provide the total number of nurses/participants in the facility and then provide the breakdown of the participants per unit (for example, 458 participants in the General ward, 390 in the pediatric ward etc.)

2) On page 8, paragraph 2, the sentence "The principal investigator was made the overall supervision..." needs to be revised.

3) On page 21, the recommendation for the Hospital administrator, bullet point i), please revise the sentence "It is better to avail of evidence-based guidelines". I believe the word 'of' should be removed.

Otherwise, the manuscript has been significantly improved.

Author response: Thank you for pointing this out. We accepted and reworked (for more, see detailed response under each point).

7. PLOS authors have the option to publish the peer review history of their article ([what does this mean?](https://journals.plos.org/plosone/s/editorial-and-peer-review-process#loc-peer-review-history)). If published, this will include your full peer review and any attached files.


If you choose “no”, your identity will remain anonymous but your review may still be made public.


**Do you want your identity to be public for this peer review?** For information about this choice, including consent withdrawal, please see our [Privacy Policy](https://www.plos.org/privacy-policy).

Reviewer #1: No

Reviewer #3: No

**Review Comments to the Authors:**

**Reviewer #3**

1. On page 6, under the heading Study design, setting, and participants, the authors need to rephrase the sentence "During the study period, there were a total of 458, 390, 254, 140 and 223". The authors should provide the total number of nurses/participants in the facility and then provide the breakdown of the participants per unit (for example, 458 participants in the General ward, 390 in the pediatric ward etc.)

Author/s response: We agreed and reworked as “During the study period, a total of 458, 390, 254, 140, and 223 nurses were working at the above-mentioned Referral Hospitals respectively. Of these, 189, 184, 128, 74, 68, and 41 were assigned in the surgical, medical, ambulatory, pediatrics, emergency, and critical care units respectively”. See in the track change document on page 6, line number 137 up to 141.

2. On page 8, paragraph 2, the sentence "The principal investigator was made the overall supervision..." needs to be revised.

Author/s response: Thank you for pointing this out. We accepted and revised it as “continuous supervision was also made by the principal investigators throughout the data collection period”. See in the track change document on page 8, line number 179 up to 181.

3. On page 21, the recommendation for the Hospital administrator, bullet point i), please revise the sentence "It is better to avail of evidence-based guidelines". I believe the word 'of' should be removed.

Author/s response: We really appreciate the issue you raised. The correction has been made accordingly. See in the track change document on page 21, line number 443.

**Again we would like to say thank you for all the given comments and your time!**
